# The effect of short-term metformin treatment on plasma prolactin levels in bromocriptine-treated patients with hyperprolactinaemia and impaired glucose tolerance: a pilot study

**DOI:** 10.1007/s12020-014-0428-2

**Published:** 2014-09-20

**Authors:** Robert Krysiak, Joanna Okrzesik, Boguslaw Okopien

**Affiliations:** Department of Internal Medicine and Clinical Pharmacology, Medical University of Silesia, Medyków 18, 40-752 Katowice, Poland

**Keywords:** Bromocriptine, Hyperprolactinaemia, Insulin resistance, Metformin

## Abstract

Metformin was found to affect plasma levels of some pituitary hormones. This study was aimed at investigating whether metformin treatment has an impact on plasma prolactin levels in bromocriptine-treated patients with hyperprolactinaemia and impaired glucose tolerance. The study included 27 patients with hyperprolactinaemia, who had been treated for at least 6 months with bromocriptine. Based on prolactin levels, bromocriptine-treated patients were divided into two groups: patients with elevated (group A, *n* = 12) and patients with normal (group B, *n* = 15) prolactin levels. The control group included 16 age-, sex- and weight-matched hyperprolactinaemia-free individuals with impaired glucose tolerance (group C).The lipid profile, fasting plasma glucose levels, the homeostatic model assessment of insulin resistance ratio (HOMA-IR), glycated haemoglobin, as well as plasma levels of prolactin, thyrotropin and insulin-like growth factor-1 (IGF-1) were assessed at baseline and after 4 months of metformin treatment (2.55–3 g daily). In all treatment groups, metformin reduced HOMA-IR, plasma triglycerides and 2-h postchallenge plasma glucose. In patients with hyperprolactinaemia, but not in the other groups of patients, metformin slightly reduced plasma levels of prolactin, and this effect correlated weakly with the metabolic effects of this drug. Our study shows that metformin decreases plasma prolactin levels only in patients with elevated levels of this hormone. The obtained results suggest that metformin treatment may bring some benefits to hyperprolactinaemic patients with coexisting glucose metabolism disturbances already receiving dopamine agonist therapy.

## Introduction

In recent years, it has become clear that elevated prolactin levels are often associated with hyperinsulinaemia, insulin resistance, atherogenic dyslipidaemia, subclinical atherosclerosis and endothelial dysfunction [1–6]. A quick-release form of bromocriptine was found to reduce plasma glucose levels, glycated haemoglobin, free fatty acids and triglycerides, and, therefore, was approved for the treatment of type 2 diabetes [7–9]. Another dopamine receptor agonist, cabergoline decreased waist circumference, plasma lipids, glycated haemoglobin, insulin and the homeostatic model assessment of insulin resistance (HOMA-IR) and this effect, observed regardless of the degree of reduction in prolactin levels, was dose-dependent [10]. Moreover, long-term cabergoline reduced the prevalence of metabolic syndrome and cardiometabolic risk associated with visceral obesity [11].

There is some evidence that metformin, considered the first-line treatment for type 2 diabetes mellitus [12] and often used in other conditions associated with insulin resistance [13], may affect pituitary hormone secretion. The drug administered to patients with untreated hypothyroidism or to hypothyroid patients effectively treated with levothyroxine reduced plasma thyrotropin levels, often to subnormal levels [14–16]. Metformin treatment of women with polycystic ovary syndrome was associated in some studies with a reduction in circulating levels of luteinizing hormone and the luteinizing hormone/follicle-stimulating hormone ratio [17–20]. Finally, in the same disorder, metformin administered alone or in combination with simvastatin, decreased circulating levels of prolactin [19–22]. Interestingly, recently we observed a patient with resistance to thyroid hormone, in whom metformin reduced plasma thyrotropin levels, probably by enhancing the effect of thyroid hormones in the pituitary [23].

However, to the best of our knowledge, prolactin-lowering effect of metformin was observed only in patients with polycystic ovary syndrome, and these patients were not treated with dopamine receptor agonists. Therefore, the aim of this study was to assess whether metformin administered to bromocriptine-treated patients with hyperprolactinaemia and impaired glucose tolerance affects plasma prolactin levels.

## Materials and methods

The participants of the study were recruited among hyperprolactinaemic subjects treated for at least 6 months with a constant dose of bromocriptine (2.5–10 mg daily). These individuals, initially diagnosed and treated in local endocrine outpatient clinics, were screened in our department for the presence of metabolic abnormalities. To be included into the study, the participants had to meet the following inclusion criteria of impaired glucose tolerance: (1) fasting plasma glucose less than 100 mg/dl and (2) plasma glucose concentration 2 h after a 75-g oral glucose load at least 140 mg/dl but less than 200 mg/dl. The exclusion criteria were as follows: mixed pituitary tumours (secreting prolactin and other pituitary hormones), macroprolactinoma, other hormonally active and non-functioning pituitary adenomas, primary hypogonadism, impaired renal or hepatic function, thyroid disorders, polycystic ovary syndrome, pregnancy or lactation, macroprolactinaemia or treatment with other drugs known to affect plasma prolactin levels. All enrolled patients provided their written informed consent for the investigation, and the study was performed according to the Declaration of Helsinki. The local ethics committee approved the study protocol. Based on prolactin levels, bromocriptine-treated patients were divided into two groups: patients with elevated (group A, *n* = 12) and patients with normal (group B, *n* = 15) circulating prolactin levels. We also recruited 16 age-, sex- and weight-matched control subjects with impaired glucose tolerance and a negative history of hyperprolactinaemia (group C). All included patients were treated with metformin. In each treatment group, the dose of this agent was gradually (over 4 weeks) increased from 0.5 g once daily to a maximum effective daily dose (2.55–3.0 g divided into three doses per day), and this final dose of metformin was administered for 4 months. Throughout the study, patients received bromocriptine at the same dose as before the beginning of the study and complied with dietary recommendations. Patients who were already taking other drugs kept their pharmacologic schedule constant during the study.

Blood samples were collected from the antecubital vein at 8 am after an overnight 12-h fasting. Moreover, glucose levels were measured in samples obtained 2 h after the oral ingestion of 75 g of glucose. To minimise analytical errors, all assays were carried out in duplicate. Plasma lipids, glucose and insulin levels were assayed by routine laboratory techniques (bioMerieux France; Beckman, Palo Alto, CA, USA; Instruments GmbH, Marburg, Germany). The homeostatic model assessment of insulin resistance (HOMA-IR) was calculated by the formula: HOMA-IR = fasting plasma glucose (mg/dl) *immunoreactive insulin (μU/ml)/405 [24]. Glycated haemoglobin was determined using DCA 2000 analyser (Bayer Ames Technicon, Tarrytown, NY). Plasma prolactin and insulin-like growth factor-1 (IGF-1) were determined by enzyme-linked immunosorbent assay (DRG Instruments GmbH, Marburg, Germany). Plasma levels of thyrotropin were measured using an electrochemiluminescence immunoassay method (Roche Diagnostics, Lewes, UK). Intra- and inter-assay coefficients of variation were less than 6.2 and 8.5 %, respectively.

The distribution of the variables was analysed using the Kolmogorov–Smirnov test. To fit a normal distribution curve, log transformation was used for the non-normal variables. Comparisons between the groups were performed using analysis of covariance followed by Bonferroni post hoc tests after consideration of age, sex, smoking, body mass index, waist-hip ratio, blood pressure, duration of bromocriptine treatment and bromocriptine dose as potential confounders. The differences between the means of variables within the same treatment group were analysed with Student’s paired *t* test. Categorical variables were analysed by *χ* ^2^test. Correlations were assessed using Kendall’s tau test. Values of *p* < 0.05 were considered statistically significant.

## Results

All treatment groups did not differ significantly in terms of demographic and anthropometric data (age, sex, smoking, body mass index, waist-hip ratio and blood pressure) (Table 1). No significant differences were observed with regard to medical background and baseline laboratory results. Expectedly, circulating prolactin levels were higher in group A than in the other groups of patients. Two patients (one from group B and one from group C) stopped participating in the study because of metformin-induced diarrhoea, nausea and increased flatulence.

**Table 1:** Baseline characteristics of patients

Variable	Bromocriptine-treated patients with hyperprolactinaemia (Group A)	Bromocriptine-treated patients with normal prolactin levels (Group B)	Control group (Group C)
Number of patients	12	14	15
Age [years; mean (SD)]	32 (3)	31 (4)	32 (4)
Women [%]	75	71	80
Smokers [%]	25	29	27
Duration of bromocriptine treatment [months; mean (SD)]	11 (2)***	12 (3)***	0 (0)
Bromocriptine dose [mg; mean (SD)]	6.4 (1.3)***	6.2 (1.2)***	0 (0)
Body mass index [kg/m^2^; mean (SD)]^a^	28.3 (2.4)	28.9 (2.2)	27.9 (2.0)
Waist-hip ratio [mean (SD)]^b^	0.92 (0.08)	0.94 (0.07)	0.91 (0.06)
Systolic blood pressure [mmHg; mean (SD)]^c^	129 (10)	127 (11)	125 (9)
Diastolic blood pressure [mmHg; mean (SD)]^c^	84 (4)	81 (3)	82 (3)
Hypertension [%]^d^	25	21	20
Prolactin-secreting tumours [%]	67***	57***	0
Fasting glucose [mmol/L; mean (SD)]	4.81 (0.25)	4.72 (0.23)	4.77 (0.21)
2-h postchallenge plasma glucose [mmol/L; mean (SD)]	9.72 (0.63)	9.17 (0.80)	9.32 (0.70)
HOMA-IR [mean (SD)]	5.0 (0.5)	4.7 (0.4)	4.6 (0.5)
Glycated haemoglobin [%; mean (SD)]	6.1 (0.2)	6.0 (0.2)	6.0 (0.2)
Total cholesterol [mmol/L; mean (SD)]	5.56 (0.61)	5.62 (0.52)	5.51 (0.58)
LDL-cholesterol [mmol/L; mean (SD)]	3.31 (0.42)	3.25 (0.32)	3.19 (0.28)
HDL-cholesterol [mmol/L; mean (SD)]	1.12 (0.18)	1.25 (0.15)	1.19 (0.16)
Triglycerides [mmol/L; mean (SD)]	2.56 (0.38)	2.43 (0.35)	2.40 (0.31)
Prolactin [ng/mL; mean (SD)]	46 (10)***^###^	12 (4)	14 (3)
IGF-1 [ng/mL; mean (SD)]	235 (61)	195 (43)	189 (41)
Thyrotropin [mIU/L; mean (SD)]	2.0 (0.7)	1.5 (0.4)	1.4 (0.5)

Only data of individuals who completed the study were included in the final analyses

^a^Weight in kilograms divided by the square of the height in meters [25]

^b^The ratio of the circumference of the waist (the midpoint between the lower margin of the last palpable rib and the top of the iliac crest) to that of the hips (the widest portion of the buttocks) [25]

^c^Average of two blood pressure measurements taken in the sitting position, spaced 2 min apart,after at least 5 min of rest [26]

^d^Blood pressure greater than 140/90 on two or more blood pressure readings taken at each of two or more visits after initial screening [26]

*** *p* < 0.001 versus control subjects (group C)

^###^ *p* < 0.001 versus bromocriptine-treated patients with normal prolactin levels (group B)

In all groups, metformin treatment led to a reduction in HOMA-IR, glycated haemoglobin, plasma triglycerides and 2-h postchallenge plasma glucose (Table 2). In patients with impaired glucose tolerance and normal prolactin levels (groups B and C), metformin produced no effect on plasma levels of the measured hormones. In patients with hyperprolactinaemia, metformin reduced plasma levels of prolactin, but did not affect circulating levels of thyrotropin and IGF-1. Between-group comparisons showed that the effect of metformin on plasma prolactin was stronger in group A than in groups B and C. However, at the end of the study, prolactin levels in group A were still higher than the remaining groups of patients (Table 2). The effect of metformin on prolactin levels in group A, as well as on 2-h postchallenge plasma glucose, glycated haemoglobin and HOMA-IR in all treatment groups tended to be more pronounced (*p* values between 0.056 and 0.096) in patients treated with 3 g than with 2.55 g of metformin daily (data not shown).

**Table 2:** The effect of metformin on glucose homeostasis markers, plasma lipids and the investigated pituitary hormones in bromocriptine-treated patients and the control group

Variable	Bromocriptine-treated patients with hyperprolactinaemia (Group A)	Bromocriptine-treated patients with normal prolactin levels (Group B)		Control group (Group C)
	Mean (SD) [∆ %]	Mean (SD) [∆ %]		Mean (SD) [∆ %]
Fasting glucose [mmol/L]				
Baseline	4.81 (0.25)	4.72 (0.23)	4.77 (0.21)	
After 4 months	4.56 (0.30) [−5]	4.51 (0.24) [−4]	4.58 (0.26) [−4]	
2-h postchallenge plasma glucose [mmol/L]				
Baseline	9.72 (0.63)	9.17 (0.80)	9.32 (0.70)	
After 4 months	7.94 (0.56) [−18]^&&&^	7.78 (0.52) [−15]^&&&^	7.72 (0.76) [−17]^&&&^	
HOMA-IR				
Baseline	5.0 (0.5)	4.7 (0.4)	4.6 (0.5)	
After 4 months	3.4 (0.4) [−32]^&&&^	3.1 (0.4) [−34]^&&&^	3.2 (0.4) [−30]^&&&^	
Glycated haemoglobin [%]				
Baseline	6.1 (0.2)	6.0 (0.2)	6.0 (0.2)	
After 4 months	5.5 (0.2) [−10]^&&&^	5.6 (0.2) [−7]^&&^	5.5 (0.3) [−8]^&&^	
Total cholesterol [mmol/L]				
Baseline	5.56 (0.61)	5.62 (0.52)	5.51 (0.58)	
After 4 months	5.38 (0.58) [−3]	5.34 (0.48) [−5]	5.28 (0.51) [−4]	
LDL-cholesterol [mmol/L]				
Baseline	3.31 (0.42)	3.25 (0.32)	3.19 (0.28)	
After 4 months	3.16 (0.38) [−5]	3.08 (0.29) [−5]	3.01 (0.26) [−6]	
HDL-cholesterol [mmol/L]				
Baseline	1.12 (0.18)	1.25 (0.15)	1.19 (0.16)	
After 4 months	1.24 (0.16) [11]	1.35 (0.18) [8]	1.30 (0.14) [9]	
Triglycerides [mmol/L]				
Baseline	2.56 (0.38)	2.43 (0.35)	2.40 (0.31)	
After 4 months	2.16 (0.28) [−16]^&^	2.06 (0.32) [−15]^&^	2.08 (0.34) [−13]^&^	
Prolactin [ng/mL]				
Baseline	46 (10)***^###^	12 (4)	14 (3)	
After 4 months	34 (8) [−26]***^###&^	11 (4) [−8]	12 (3) [−14]	
IGF-1 [ng/mL]				
Baseline	235 (61)	195 (43)	189 (41)	
After 4 months	220 (62) [−6]	185 (40) [−5]	176 (29) [−7]	
Thyrotropin [mIU/L]				
Baseline	2.0 (0.7)	1.5 (0.4)	1.4 (0.5)	
After 4 months	1.6 (0.6) [−20]	1.3 [−13]	1.2 [−14]	

Only data of individuals who completed the study were included in the final analyses

*** *p* < 0.001 versus control subjects (group C)

^###^ *p* < 0.001 versus bromocriptine-treated patients with normal prolactin levels (group B)

^&^ *p* < 0.05

^&&^ *p* < 0.01

^&&&^ *p* < 0.001 versus baseline value

At entry, prolactin levels correlated weakly with HOMA-IR, plasma triglycerides and 2-h postchallenge plasma glucose (*r* values between 0.22 [*p* < 0.01] and 0.32 [*p* < 0.001]). In patients with hyperprolactinaemia, but not in groups B and C, the effect of metformin on circulating prolactin levels correlated with baseline prolactin levels (*r* = 0.51, *p* < 0.001), as well as weakly with the effect of this drug on HOMA-IR (*r* = 0.34, *p* < 0.001), 2-h postchallenge plasma glucose (*r* = 0.25, *p* < 0.01) and triglycerides (*r* = 0.29, *p* < 0.01). The effect of metformin on glucose homeostasis markers, plasma lipids and the investigated pituitary hormones correlated neither with the duration of bromocriptine treatment nor with bromocriptine dose.

## Discussion

The major finding of this study is that metformin reduced prolactin levels only in patients with hyperprolactinaemia, while this effect was not observed in subjects with normal prolactin levels. Considering the exclusion criteria (liver and renal failure), our results indicate that the impact of metformin on circulating levels of this hormone is noticeable only if its pituitary production is increased. In line with this hypothesis, metformin did not change plasma levels of thyrotropin and IGF-1, the baseline levels of which were within the reference range.

Although higher than in the Diabetes Prevention Programme (1.7 g daily) [27], metformin dose in our study was similar to the maximum dose of metformin (2.55 g daily), used by more than half of patients in the United Kingdom Prospective Diabetes Study, the largest clinical research study into diabetes ever conducted at the time [28]. At the same doses as in the present study, metformin, administered because of coexistent type 2 diabetes, reduced androgen levels in women with non-classic congenital adrenal hyperplasia [29] as well as thyrotropin and thyroid hormone levels in a patient with resistance to thyroid hormone [30]. Also in patients with impaired glucose tolerance, high-dose metformin treatment was well tolerated and produced multidirectional pleiotropic effects [31, 32], while in individuals with insulin resistance it produced the strongest effect on glycaemic control and plasma lipids [33]. Interestingly, the impact of metformin on prolactin levels, as well as on glucose homeostasis was a bit stronger if administered at higher (3 g) than lower (2.55 g) daily dose, suggesting that this effect is dose-dependent. The difference, however, did not reach the level of significance, probably because of a small number of patients treated with each dose.

It should be stressed that the prolactin-lowering effect in patients ineffectively treated with bromocriptine was at most moderate and post-treatment prolactin levels still exceeded normal values. Taking into account that dopamine agonists are the drugs of choice in the treatment of elevated prolactin levels [34], and markedly reduce cardiometabolic risk in hyperprolactinaemic patients [7–11], it seems that a proper bromocriptine dose adjustment or replacing bromocriptine with cabergoline in Group A patients would have produced greater effects on prolactin levels and on metabolic parameters than metformin addition. Therefore, metformin should be rather reserved for the treatment of coexisting glucose metabolism abnormalities and eventually of hyperprolactinaemia if they are not reversed by dopamine agonist therapy. Our results also suggest that in normoprolactinaemic patients treated with bromocriptine, the dose of this drug does not require a reduction, if a patient has to be treated with metformin because of coexisting glucose metabolism abnormalities.

We included individuals with concomitant impaired glucose tolerance, because from a cardiometabolic point of view these patients seem good candidates for metformin treatment. In the Diabetes Prevention Programme [27] and a Chinese study [35], metformin decreased the incidence of type 2 diabetes in subjects with impaired glucose tolerance, only some of whom had coexistent impaired fasting glucose. Moreover, the investigated group of patients is probably particularly prone to the earlier development and faster progression of atherosclerosis. Hyperprolactinaemia is associated with preclinical atherosclerosis, while patients with hyperprolactinaemia might experience cardiovascular disease in the long term [36]. Impaired glucose tolerance is characterised by a greater risk of cardiovascular disorder than isolated impaired fasting glucose. After adjusting for hypertension and dyslipidaemia, only impaired glucose tolerance remains an independent risk factor of cardiovascular events or death [37]. Moreover, the association between impaired glucose tolerance and diabetes development is better documented than for impaired fasting glucose [13]. Therefore, although the effect on prolactin levels was relatively mild and limited to patients with elevated prolactin levels, metformin may be useful in hyperprolactinaemic patients at high risk of diabetes and cardiovascular disease. Unfortunately, the study protocol does not allow us to conclude whether metformin produces a similar effect in hyperprolactinaemic patients with no evidence of hyperinsulinaemia, glucose abnormalities and metabolic syndrome.

It cannot be excluded that metformin treatment may also bring some benefits to hyperprolactinaemic patients failing to achieve normal prolactin level on maximally tolerated doses of dopamine agonist, who are poor operative candidates or decline surgery. This question requires, however, further studies. We excluded individuals with macroprolactinoma who, because of potentially aggressive tumour behaviour, should be treated with cabergoline rather than with bromocriptine [38]. Moreover, in patients with large prolactinomas, the initial prolactin level may be read erroneously as normal or only mildly elevated (“the hook effect”) [39]. Finally, large prolactin-secreting pituitary tumours often (even in 78 % of patients) lead to the development of hypopituitarism; the gonadal and somatotropic axes being the most frequently affected [40]. The eventual presence and severity of secondary hypogonadism and other manifestations of hypopituitarism might have affected our findings.

The obtained results may be theoretically attributed to a time-dependent effect of bromocriptine treatment but this explanation seems much less probable. Eligible patients were enrolled in the study only if they were treated with a constant dose of bromocriptine for at least 6 months (on average for 11 months) before the study onset and no changes in dosage were allowed during the entire study period. A reduction in circulating prolactin levels was observed in a subgroup of patients in whom hyperprolactinaemia was unrelated to prolactinoma and, therefore, could not be associated with a decrease in tumour size and/or tumour apoplexy. Finally, previously we followed 11 patients meeting the inclusion criteria of group A but not receiving metformin, in whom, despite elevated prolactin levels, bromocriptine treatment was continued in the same dose. In this group of patients, comparable to group A with respect to age, sex, body weight and bromocriptine dose, 6 months later, circulating prolactin levels did not differ from baseline values [Krysiak et al., unpublished observations].

Another interesting question is metformin use in hyperprolactinaemic subjects not treated with dopamine agonists. Our study indirectly suggests that metformin may decrease prolactin levels also in this group of patients because its impact on circulating prolactin levels correlated with baseline prolactin levels, but not with the duration and dosage of bromocriptine treatment. This interesting hypothesis will be verified in our future research.

The study protocol does not allow us to unequivocally explain the mechanism responsible for this action of metformin. Metformin may change the affinity and/or the number of dopamine receptors or of receptors for other compounds regulating production, secretion and metabolism of prolactin, may enhance gastrointestinal absorption and/or metabolism of bromocriptine, as well as may directly affect prolactin pharmacokinetics. Interestingly, animal studies carried out in our laboratory evidenced that metformin penetrates the blood–brain barrier, and its content in the pituitary is higher than in any other brain structure [41]. In the light of these results, it seems that the pituitary is an important target for metformin action and that the prolactin-lowering effect of this agent results, at least in part, from its action at the level of pituitary lactotropes. Taking into account that this drug was found to reduce plasma levels of other pituitary hormones [14–18, 23], it may be assumed that metformin affects the function of different types of pituitary cells, provided their secretory function is enhanced. In agreement with this hypothesis, in the study by Cappelli et al. [14], the authors did not find any changes in plasma thyrotropin levels in metformin-treated patients with normal hypothalamic-pituitary-thyroid axis activity. Although we cannot totally exclude the possibility that metformin affects pharmacokinetics of bromocriptine, this explanation is much less likely. In our study, this drug did not reduce plasma prolactin levels in group B, treated like group A, with bromocriptine. Moreover, metformin was found to reduce, not to improve, absorption of vitamin B_12_, folates, amino acids, glucose and some drugs [42]. The obtained results cannot be also explained by a reduction in circulating levels of luteinizing hormone and the luteinizing hormone/follicle-stimulating hormone ratio, as demonstrated in patients with polycystic ovary syndrome [19, 20], because this syndrome belonged to the exclusion criteria, while microprolactinoma, traumatic brain injury and primary empty sella syndrome responsible for hyperprolactinaemia in our study are characterised by normal or reduced gonadotropin levels [38, 39].

Metformin action on prolactin in group A correlated with the degree of a reduction in HOMA-IR, 2-h postchallenge plasma glucose and triglycerides. Although the aforementioned correlations were weak, their presence suggests that some signal pathways regulating prolactin production and release, and signal pathways affected by insulin may overlap to some extent. The complex regulation of prolactin secretion and release, involving both direct and indirect mechanisms [43, 44] and their mutual interactions may explain why these correlations were absent in normoprolactinemic patients.

This study has some limitations. The most important of them is a small number of participants and the short duration of treatment, limiting the statistical significance of the findings. Moreover, all hyperprolactinaemic patients received bromocriptine. It cannot be completely excluded that the effect of metformin treatment on plasma prolactin levels is different in patients with elevated prolactin levels receiving other dopaminergic agents and/or, as mentioned above, in non-treated patients. Finally, all our patients had coexistent impaired glucose tolerance. Therefore, the question whether a similar effect is observed in patients with other glucose metabolism abnormalities (type 2 diabetes and impaired fasting glucose), or in patients with normal glucose tolerance remains still open.

To sum up, this study shows that metformin slightly reduces elevated prolactin levels in patients chronically treated with bromocriptine. This effect, correlating weakly with metabolic effects of bromocriptine, suggests that metformin may be useful in the management of hyperprolactinaemic patients with coexisting glucose metabolism disturbances already receiving dopamine agonist therapy.

## Abbreviations

HDL: High-density lipoprotein
HOMA-IR: The homeostatic model assessment of insulin resistance ratio
IGF-1: Insulin-like growth factor-1
LDL: Low-density lipoprotein
SD: Standard deviation
